# The biological behavior of tRNA-derived fragment tRF-Leu-AAG in pancreatic cancer cells

**DOI:** 10.1080/21655979.2022.2064206

**Published:** 2022-04-20

**Authors:** Shizhen Sui, Zhihuai Wang, Xiaohan Cui, Lei Jin, Chunfu Zhu

**Affiliations:** a Graduate School of Dalian Medical University, Dalian Medical University, Dalian, Liaoning, China; b Department of Hepatobiliary Surgery, The Affiliated Changzhou No. 2 People’s Hospital of Nanjing Medical University, Changzhou, Jiangsu, China

**Keywords:** Pancreatic cancer, tRNA-derived fragments, UPF1

## Abstract

Pancreatic cancer (PC) is a life-threatening cancer with increasing incidence in developed countries. Reports indicate that tRNA-derived fragments (tRFs) are possible therapeutic targets and biomarkers for cancer treatment. Nonetheless, the effect of tRF-Leu-AAG on PC is unclear. This study aims to explore the role of tRF-Leu-AAG and upstream frameshift mutant 1 (UPF1) in the development of PC and its potential underlying mechanisms. High-throughput second-generation sequencing techniques were used to detect the expression of tRFs in cancerous and adjacent normal tissues from PC patients. The role of tRF-Leu-AAG proliferation in PC cells was investigated via the Cell Counting Kit-8 (CCK8) assay. The effect of tRF-Leu-AAG on the invasion and migration ability of PC cells was also determined by the transwell assay. Thereafter, the downstream target genes of tRF-Leu-AAG were comprehensively predicted using bioinformatics analysis databases. We also used the Dual-Luciferase Reporter assay to assess the nexus between tRF-Leu-AAG and UPF1. Eventually, Western Blot was used to validate the expression of UPF1 in PC cells. A total of 33 tRF expressions significantly varied from PC patients. RT-qPCR confirmed that the expression of tRF-Leu-AAG was observably up-regulated in PC cells as compared to the control cells. Importantly, knockdown of tRF-Leu-AAG observably inhibited cell proliferation, migration, and invasion. Furthermore, according to the predicted frameshift database results, the UPF1 acted as downstream target genes for tRF-Leu-AAG and significantly down-regulated UPF1 expression.

## Graphical Abstract

**Figure:**
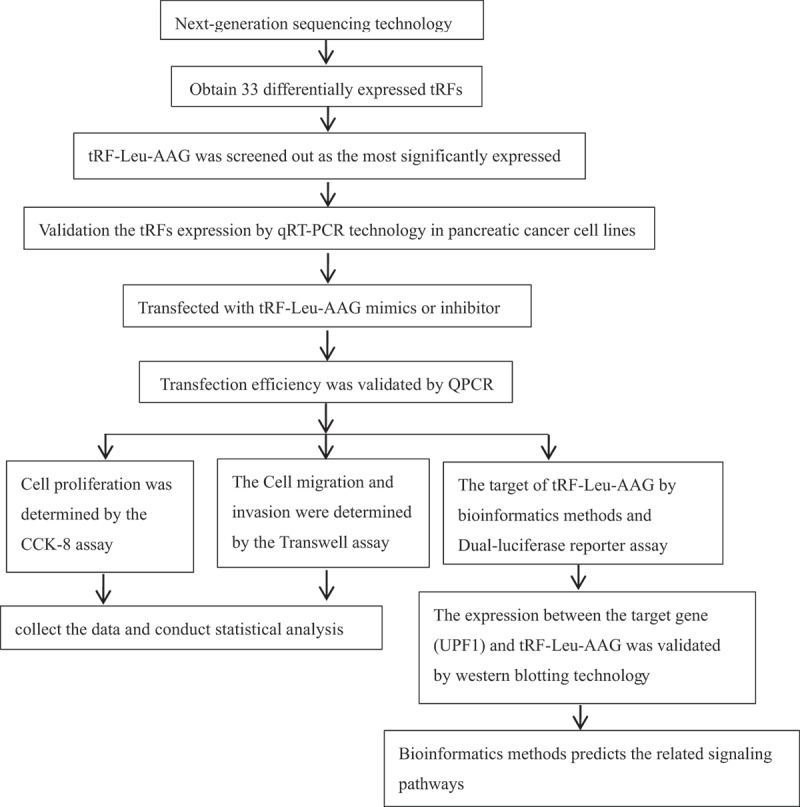

## Introduction

Pancreatic cancer (PC) is a fatal cancer with 5-year survival rate of about 8% [1]. PC has an insidious onset, and its early stage lacks specific symptoms. Besides, its median survival period of fewer than 11 months and the prognosis are extremely poor. This is the fourth leading cause of death from cancer in the world [2]. So far, PC lacks a specific peripheral blood screening method.

With the advent and application of high-throughput second-generation sequencing and microchip technologies, a novel class of sncRNAs has been found from tRNAs [3]. These ncRNA are identified as tRNA fragments (tRNA-derived fragment, tRFs) [4]. On the basis of their relative length and biogenesis, tRFs are classified into five principal subcategories, i.e., tRF-5, tRF-3, tRF-2, tRF-1, and i-tRF [5]. Notably, tRF^Ala^, tRF^Glu^, tRF^Tyr^, and tRF^Asp^ were the first tRNA fragments to inhibit breast cancer progression. Specifically, they inhibit multifarious oncogenic transcripts by replacing the 3ʹ UTR of YBX1. Reports have also shown that TRFs can restrain cell growth, cell migration, and invasion [6]. Recent studies have identified tRFs as novel potential targets and biomarkers for cancer treatment [7]. Nonetheless, the role of tRF-Leu-AAG in PC remains unclear.

Herein, we investigate the roles of tRF-Leu-AAG in PC cells. Cancerous and adjacent normal tissues from PC patients were analyzed using high-throughput second-generation sequencing techniques. Thereafter, the impact of tRF-Leu-AAG on PC cells was validated by Transwell and CCK-8. Furthermore, target genes of tRF-Leu-AAG were predicted through bioinformatics analysis and then tested using Western Blot analysis and Dual-Luciferase Reporter gene assays to verify their nexus.

In summary, this study suggests that tRF-Leu-AAG may promote cell proliferation and migration by regulating UPF1 in PC. We will verify it by cell phenotypic experiments. The detection of tRF-Leu-AAG may provide new ideas for the early diagnosis of PC.

## Materials and methods

### 1. Study population and sample collection

Three pairs of cancerous and adjacent normal tissues collected from PC patients attending Changzhou No. 2 People’s Hospital were used for total RNA-seq. The patients characteristics are presented in Table S1. Exclusion criteria included patients with a previous history of any cancer, metastatic cancer at other sites, or those who underwent chemotherapy or radiation therapy, with diseases including hypertension, diabetes, and infectious diseases. After signing the informed consent form, each participant was interviewed to extract their demographic and lifestyle information, including age and gender. The Ethics Committee of the Affiliated Changzhou No. 2 People’s Hospital of Nanjing Medical University (Changzhou, China) approved this study.

### 2. High-throughput second-generation sequencing

Total RNA of extracted samples or purified sRNA fragments were extracted and reverse transcribed into cDNA followed by PCR amplification. Subsequently, the glue fragment libraries were recovered. Meanwhile, the qualified libraries were sequenced by machine. The raw data (raw reads) obtained from Illumina HiSeqTM 2500 sequencing were first filtered: first, the two-end structures of the reads were cut out; second, removed low-quality and fragment length <15 nt of the reads; and third, preliminary filtering of data and the high-quality data (clean reads) were obtained. The identified tsRNA was subjected to expression calculation, tsRNA expression clustering, and differential expression tsRNA analysis between samples. It was eventually sequenced using Illumina NextSeq 500 (#FC-404-2005, Illumina).

### 3. Bioinformatics analysis

Two groups of atlas differences were in comparison (e.g. disease and control). A ‘fold change’ was calculated between each tRF/tiRNA group using the normalized label number of the tRNA exegesis in the GtRNAdb, including label counts for each sample. tRFs/tiRNAs with fold changes ≥2 and *P*-value ≤ 0.05 were selected as the observably differentially expressed tRFs/tiRNAs. The target genes of tRF-Leu-AAG were predicted by TargetMiner (http://www.isical.ac.in/~bioinfo_miu/targetminer20), TargetScan (http://www.targetscan.org/vert_70/), and TargetRank (http://hollywood.mit.edu/targetrank/) databases.

### 4. Cell culture

This work used BxPC-3, PANC-1, and ASPC-1 (Shanghai Zhongqiaoxinzhou Biotech) as well as normal human pancreatic HPDE6-C7 (Shanghai Qincheng Biotech) cell lines. The PANC-1 cell was cultured in Dulbecco’s Modified Eagle’s Medium (DMEM), whereas ASPC-1, BxPC-3, and HPDE6-C7 cells were cultured in RPMI 1640 medium, respectively, supplemented with 10% fetal bovine serum (FBS, Gibco, 10,099-141, AU) and 1% penicillin–streptomycin (P/S, Gibco, 15,140-122, US), incubated in a 37°C, 5% CO_2_ incubator.

### 5. Cell transfection

The cells were added into six-well plates at a density of 2 × 10^5^ cells/ml per well and cultured with a complete medium for 24 h until the confluence reached 70–80%. Subsequently, the cells were transfected with tRF-Leu-AAG mimics or inhibitors based on the instructions of Lipofectamine^TM^ 3000 (Invitrogen, CA, USA) and then cultured for 24 h and kept for subsequent experiments.

### 6. qRT-PCR assay

TRIzol reagent (Invitrogen, Carlsbad, CA, USA) was used to extract total RNA in cells. Reverse transcription was executed as per ribo*SCRIPT*^TM^ Reverse Transcription Kit (Guangzhou Ruibo Biotechnology Co., Ltd., China) instructions. The primers (U6 F, U6 R, tRF-Leu-AAG F, tRF-Leu-AAG R) (Table 1) were obtained from Guangzhou Ruibo Biotechnology Co., Ltd., China. A microplate reader (BioTek, Epoch, USA) was used to detect the concentration of extracted RNA. qRT-PCR was performed based on Bulge-Loop^TM^ miRNA qRT-PCR Primer (Guangzhou Ruibo Biotechnology Co., Ltd., China) instructions. The 2^–∆∆Ct^ method was using to calculate relative gene expression.

**Table 1.** Sequences of primer and plasmid

Gene Name	Sequence (5’–3’)
GAPDH F	TGAAGGTCGGAGTCAACGGATTTGGT
GAPDH R	CATGTGGGCCATGAGGTCCACCAC
tRF-Leu-AAG mimics	ATCCCACCGCTGCCACCA
tRF-Leu-AAG inhibitor	UGGUGGCAGCGGUGGGAU
NC-mimics	UUUGUACUACACAAAAGUACUG
NC-inhibitor	CAGUACUUUUGUGUAGUACAAA

### 7. Cell counting kit-8 (CCK-8) assay

Cells were harvested 48 h after transfection and then seeded with 2 × 10^4^ cells/ml of cells per well in 96-well plates. The experimental group and control group had 5 replicates, then the 96-well plates was maintained in the incubator for 24, 48, 72, and 96 h. The cells were cultivated for 2 h with 10 µl CCK-8 (Beyotime, Shanghai, China) solution in each well. The concentration below 450 nm absorbance of cells was measured by the microplate reader (BioTek, Epoch, USA).

### 8. Transwell assays

Cells in the log growth period were selected and replaced with a serum-free medium to keep the cells under starvation for 6 h. Previously, 50 mg/L of Matrigel glue was diluted at a ratio of 1:8 with a serum-free medium, and then it was added into the bottom of the upper chamber, air-dried at room temperature, and hybridized with culture medium before use. The cells were then digested with trypsin, and digestion was terminated with a complete medium containing 10% FBS. Cells were centrifuged at 10,000 rpm for 5 min, and then the supernatant was discarded, washed twice in PBS, and resuspended with a serum-free medium. After cell count, planting 5 × 10^4^ cells in upper chambers containing with matrix gel and upper chamberswithout matrix gel, both in a volume of 500 µl. Three samples were repeated. The chamber was placed into 24-well plates, and 700 µl of complete medium containing 10% FBS was added into the lower chamber. Cell cultures were then incubated at 37°C and 5% CO_2_ for 24 h. Thereafter, the chamber was removed, placed into new wells, and washed twice in PBS. Cotton swabs were then wiped off the upper chamber and fixed in 800 µl of 4% paraformaldehyde for 15 min. After being naturally air-dried, the upper chamber was stained with 800 µl of 0.5% crystal violet for 20 min, washed three times in PBS, and allowed to dry. Furthermore, cells moving to the lower microporous membrane were counted under an inverted microscope, and four randomly taken visual fields for each sample were averaged.

### 9. Western blotting

Cells were harvested, supplemented with cell lysate, and total proteins were extracted using a cell lysis buffer for Western blotting (Beyotime, Biotechnology, P0013J, China). Then, a microplate reader (BioTek, Epoch, USA) was used to determine the concentration of protein. Sodium dodecyl sulfate-polyacrylamide gel electrophoresis (SDS-PAGE) of 10% concentration was used to load on equal amounts of proteins in each set for separating the proteins. The polyvinylidene fluoride (PVDF) membrane was then used to transfer these proteins. Subsequently, the proteins were blocked with a quick-blocking solution for 30 min. UPF1 primary antibody (1:1000; 23,379-1-AP, Proteintech) or β-Actin primary antibody (1:5000; 60,008-1-Lg, Proteintech) was overnight incubated at 4°C. They were rinsed three times using Tris-buffered saline with Tween (TBST) for 10 min each. Thereafter, the secondary antibodies were maintained at room temperature for 1–2 h. Then, 200 µl of Electrochemiluminescence (ECL) reagent was dropped to the protein band. Finally, protein bands were photographed using the chemiluminescent gel imaging system (FluorChem Q, Protein Simple, USA).

### 10. Dual-luciferase reporter gene assay

Wild-type and mutant UPF1 were constructed and then co-transfected with tRF-Leu-AAG mimic/negative control and wild-type/mutant UPF1 into cells. Dual-Luciferase Reporter gene assay kit (Promega, Madison, WI, USA) was used to detect the activity of cell luciferase.

### 11. Statistical analysis

Each experiment was repeated three times. Two groups of data were analyzed by Student's t-test. One-way ANOVA was used to analyze multiple sets of data. A *P*-value of less than 0.05 was considered statistically significant (* *P* < 0.05, ** *P* < 0.01, and *** *P* < 0.001).

## Results

In this study, we obtained 33 differentially expressed tRFs by high-throughput sequencing of cancerous and adjacent tissues from three PC patients. Three tRFs with significant differences were selected and verified by qRT-PCR, resulting in the most distinct tRF-Leu-AAG. We speculated that tRF-Leu-AAG may have an effect on cell proliferation, migration, and invasion, so we performed CCK-8 and Transwell assays. To further explore the influence mechanism of tRF-Leu-AAG, we performed the target gene prediction and verified their relationship. Besides, the possible pathways between tRF-Leu-AAG and target genes were explored by functional enrichment analysis.

### 1. Small RNA distribution and abundance

Tissue samples from three PC patients were performed by high-throughput second-generation sequencing [8] to identify tRFs associated with PC. After controlling the quality of the Illumina, the sequencing reads were trimmed using 5ʹ, 3ʹ-adapters, filtered for ≥15 nt using cropping software; then, the mature tRNA and anterior tRNA sequences of GtRNAdb were aligned using Novo Align software (v2.07.11). The remaining reads were aligned to the transcriptome sequence. The statistical information of the read is listed in Figure S1. Six types of tRF including tiRNA-5, tiRNA-3, i-tRF, tRF-5, tRF-3, and tRF-1 were found in PC patients, out of which tiRNA-5 was the dominant tRF (25.9%), followed by tRF-3 (22.8%) and i-tRF (13.8%).

### 2. Differential gene expression analysis

**Figure 1.**
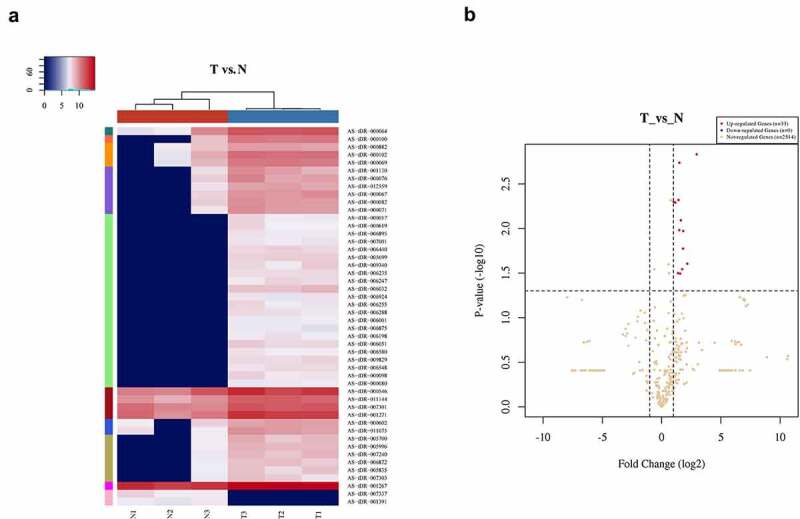
Differentially expressed tRFs in PC patients. (a) Heat map of gene expression data obtained from the cancerous and adjacent normal tissues; it was performed using the genes that between 0.5-0.85 quantile coefficient of variation (CV) based on TPM counts (T vs N). The expression levels above the mean are indicated in red. Blue indicates the expression levels below the mean. The colored bars on the right side of the panel indicate the 10 partitions performed using K-means, while the colored bars on the top panel show the sample groups. (b) Volcano plot for T vs N. Differentially expressed tRF and tiRNAs are indicated in red and green (red represents up-regulated, green represents down-regulated), with absolute fold change values greater than 2 being statistically significant. Non-differentially expressed tRF and tiRNAs are indicated in gray, whether FC or *P*-values are satisfied.

**Figure 2.**
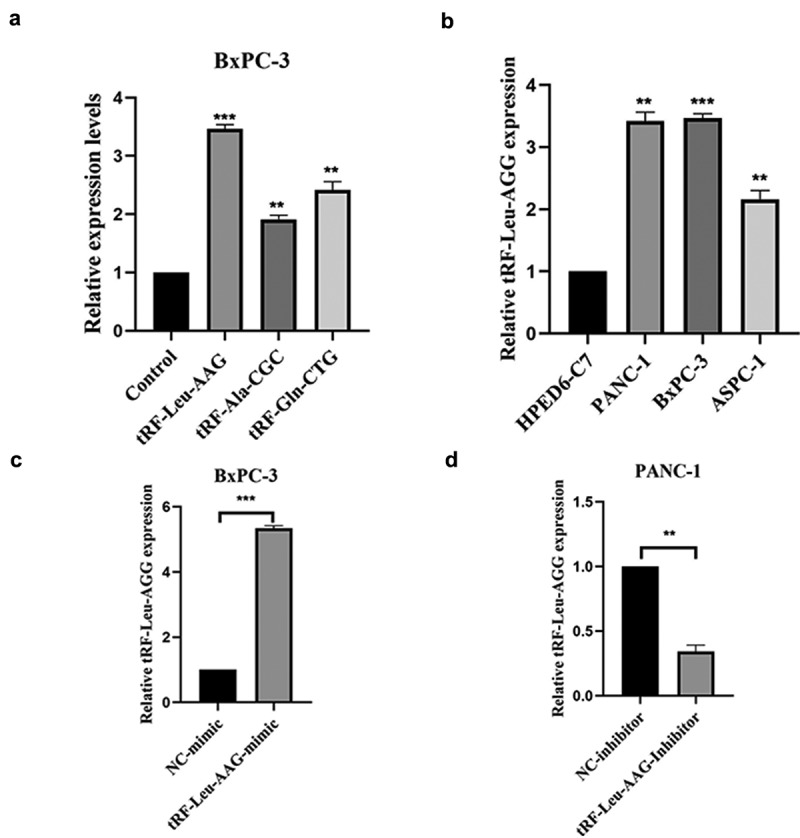
(a and b) qRT-PCR was used to measure the expression of tRF-Leu-AAG in PC cells and normal cells; t-test. Validation of the differentially expressed tRFs. (c and d) qRT-PCR was used to measure the expression of tRF-Leu-AAG in BxPC-3 cells transfected with the tRF-Leu-AAG mimics or PANC-1 cells transfected with the tRF-Leu-AAG inhibitor; t-test. * *P* < 0.05, ** *P* < 0.01, and *** *P* < 0.001.

There were 2547 tRFs expressed in tissues from PC patients. Out of these, 33 differentially expressed tRFs were up-regulated while none were down-regulated in PC patients (Figures 1(a, b)). To verify the differential expression based on fold change and abundance, real-time PCR was used to evaluate three highly expressed candidate tRFs, i.e., tRF-Leu-AAG, tRF-Ala-CGC, and tRF-Gln-CTG, in BxPC-3 and HPDE6-C7 cells. Among them, the up-regulation of tRF-Leu-AAG was the most significant (*P*< 0.001) (Figure 2(a)). Furthermore, tRF-Leu-AAG was evaluated in PC cells (PANC-1, BxPC-3, and ASPC-1) using RT-PCR. Consequently, tRF-Leu-AAG expression was observably higher in BxPC-3 and PANC-1 cells than in ASPC-1 cells (Figure 2(b)). After the transfection of tRF-Leu-AAG-mimics, the tRF-Leu-AAG expression was observably up-regulated in BxPC-3 cells relative to the cells transfected with NC-mimics (Figure 2(c)). Then, tRF-Leu-AAG expression was observably down-regulated in the PANC-1 cells transfected with the tRF-Leu-AAG inhibitors compared to those transfected with the NC-inhibitors (Figure 2(d)).

### 3. Knockdown of tRF-Leu-AAG inhibits the proliferation, migration, and invasion of pancreatic cancer cells

**Figure 3.**
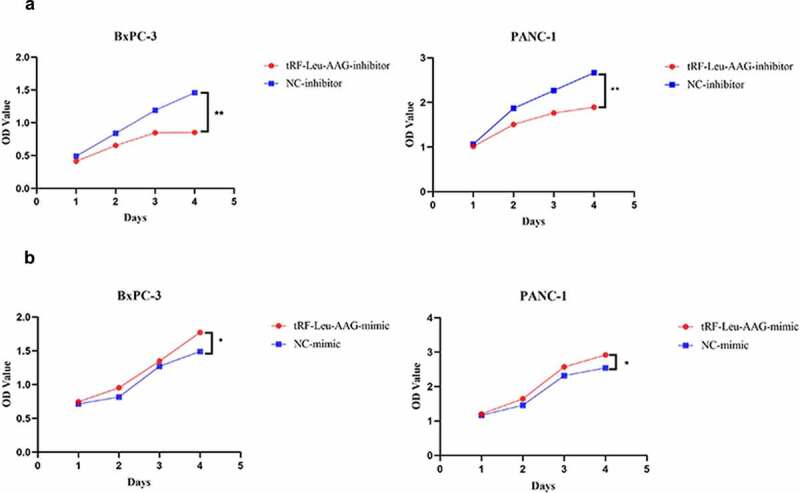
The proliferation of PC cells was suppressed after knockdown of tRF-Leu-AAG.

**Figure 4.**
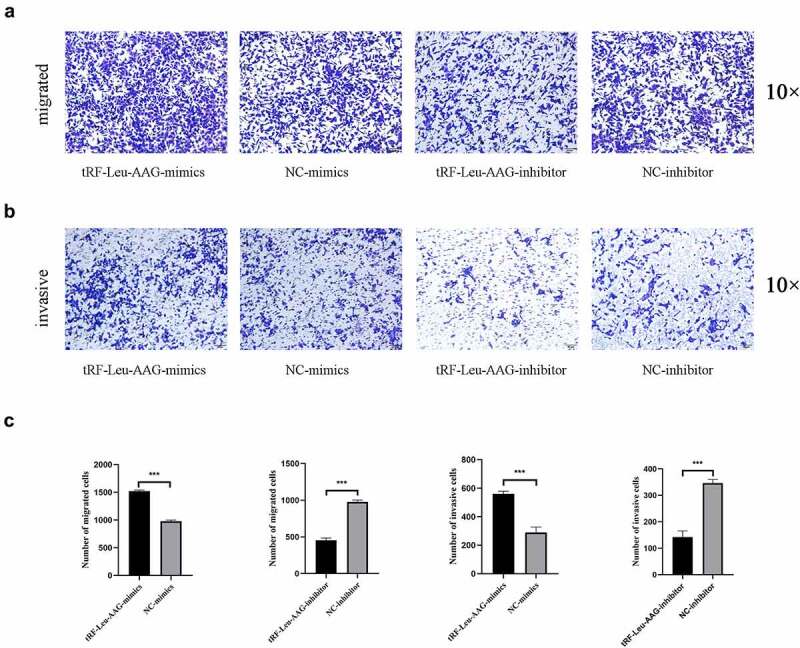
The migration and invasion of PC cells were inhibited while knockdown of tRF-Leu-AAG. (a–c). More cells migrated and invaded in the mimics group than in the inhibitor group * *P* < 0.05, ** *P* < 0.01, and *** *P* < 0.001.

CCK-8 assay was used to evaluate the effect of tRF-Leu-AAG on the proliferation of BxPC-3 and PANC-1. It is shown that unlike the control group, the viability of BxPC-3 and PANC-1 cells is observably inhibited after tRF-Leu-AAG knockdown (Figure 3(a)). In contrast, the vitality of BxPC-3 and PANC-1 cells was remarkably increased after overexpression of tRF-Leu-AAG (Figure 3(b)). In addition, we determined the effect of tRF-Leu-AAG on migration and invasion capacities of BxPC-3 cells using Transwell assay. As a consequence, the number of cells with migration and invasion transfected with the tRF-Leu-AAG inhibitor group was observably reduced compared to that in the control group (Figure 4(a)), whereas compared to that in the control group, the number of cells with migration and invasion transfected with the tRF-Leu-AAG mimics group was observably increased. (Figure 4(b)).

### 4. tRF-Leu-AAG promotes pancreatic cancer development via suppressing UPF1

**Figure 5.**
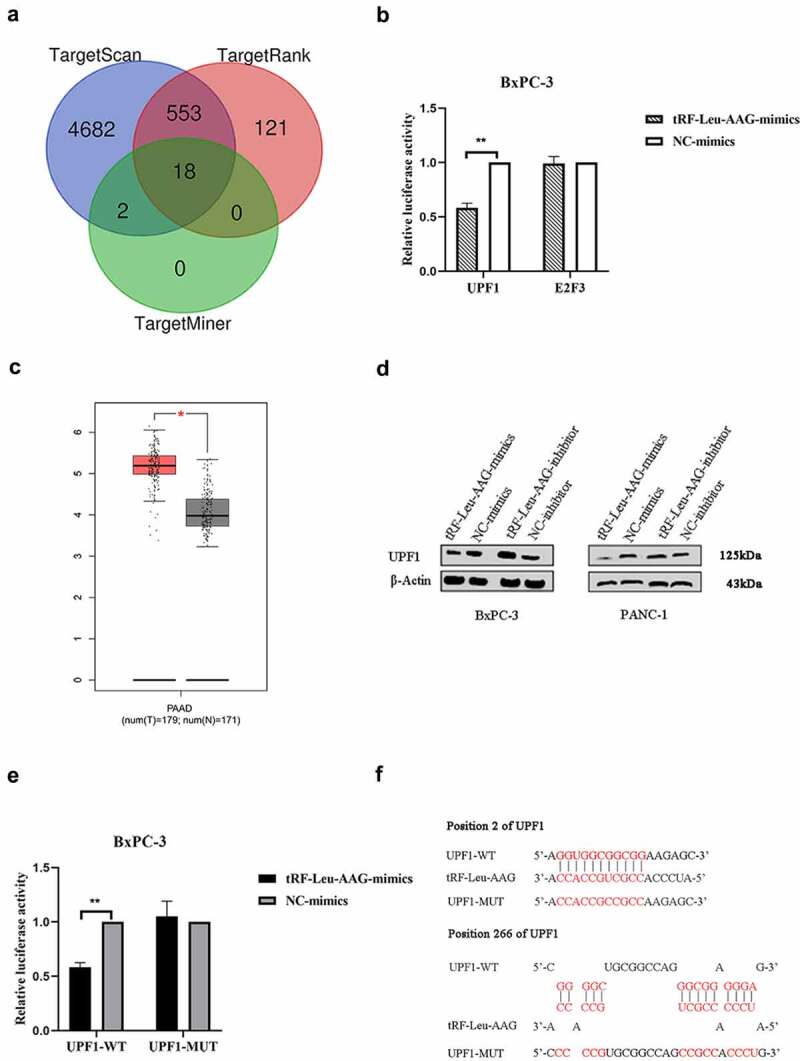
The tRF-Leu-AAG promoted PC development by suppressing UPF1.

Three mRNA target prediction databases including TargetMiner, TargetRank, and TargetScan based on the binding targets in the 3ʹUTR were used to identify and select the downstream targets of the tRF-Leu-AAG. Among the 18 genes overlapping in these databases (Figure 5(a)), two genes (E2F3 and UPF1) associated with cell proliferation were selected. To confirm the nexus between the predicted target genes and tRF-Leu-AAG, the 3ʹUTR of the target gene was cloned into a dual-luciferase UTR vector. Significantly, tRF-Leu-AAG inhibited the 3ʹUTR of UPF1 particularly (Figure 5(b)). The mRNA with differential expression was analyzed by the Limma software package of the R software (version: 3.40.2). Adjusted *P*-values were analyzed in either Genotype-Tissue Expression (GTEx) or The Cancer Genome Atlas (TCGA) [9,10] to correct for the false-positive results. ‘The absolute value of the log2 (fold change) is greater than 1 and adjusted *P* < 0.05’ was defined as a screen for differential expression of the threshold mRNA (Figure 5(c)). At the protein level, over-expression of tRF-Leu-AAG significantly down-regulated the expression of UPF1 in BxPC-3 and PANC-1 cells. In contrast, the UPF1 levels in both BxPC-3 and PANC-1 cells were up-regulated during down-regulation of tRF-Leu-AAG (Figure 5(d)). By evaluating the 3ʹUTR sequence of UPF1, we found two perfectly matched binding sites to the tRF-Leu-AAG sequence. Then, mutations were generated at the binding site to eliminate the tRF-Leu-AAG-UPF1 3ʹUTR interaction (Figure 5(e)). As expected, the reporter gene carrying the intact UPF1 3‘UTR was effectively inhibited by tRF-Leu-AAG, while the UPF1 3ʹUTR carrying the mutant binding site was resistant to suppression by tRF-Leu-AAG (Figure 5(f)). As shown, UPF1 protein levels negatively correlated with tRF-Leu-AAG levels in PC cell lines. Taken together, the tRF-Leu-AAG forthright regulates UPF1 expression via the binding sites in the 3ʹUTR.

### 5. Functional enrichment analysis of tRF-Leu-AAG target genes

**Figure 6.**
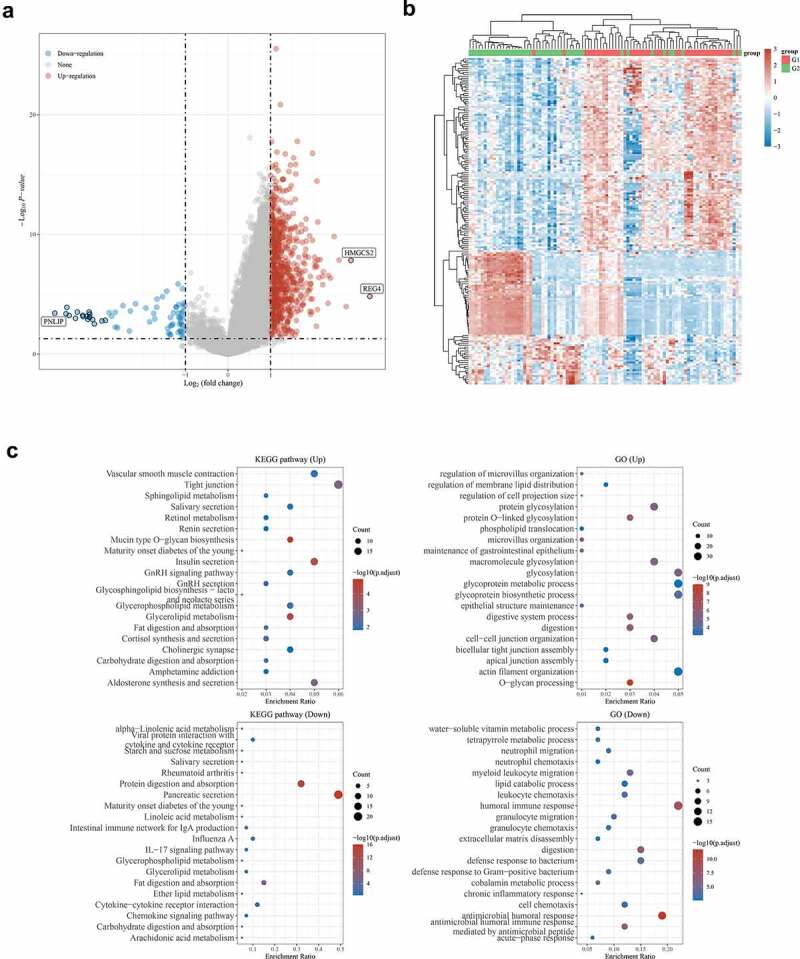
Functional enrichment analysis of tRF-Leu-AAG target genes.

We used functional enrichment analysis to analyze the data to further confirm the functions of target genes. Volcano plots were constructed using fold-change values and adjusted P. The red dots in the figure indicate over-expressed mRNAs, while the blue dots indicate down-expressed mRNAs, which are statistically significant (Figure 6(a)). Hierarchical clustering analysis of mRNAs was differentially expressed between cancerous and adjacent normal tissues (Figure 6(b)). Enriched Kyoto Encyclopedia of Genes and Genomes (KEGG) signaling was selected to demonstrate the main biological role of the primary underlying mRNA. The abscissa is the gene ratio, whereas the enrichment pathway is the ordinate. Gene Ontology (GO) is a widely-used tool for annotating genes with functions [11]. The R software (version 3.18.0) with ClusterProfiler package was used to perform the clustering of biological processes (BP), molecular functions (MF), and cellular components (CC) of potential targets. Among the enrichment results, FDR < 0.05 or *P* < 0.05 was considered a meaningful pathway for enrichment (Figure 6(c)).

## Discussion

Whilst acknowledging the recent improvements in the field of diagnosis and treatment of cancer, PC remains a life-threatening cancer [12]. Its incidence has been increasing in recent decades. PC is characterized by early relapse and metastasis, as well as resistance to radiotherapy and chemotherapy [13]. Moreover, 85% of patients miss the chance for surgery, as most patients are already in an advanced stage once detected [14]. Early diagnosis is most urgently needed because of the characteristics of PC [15]. Besides, more efficient molecular medical approaches including miRNA and tRF are necessary. However, whether tRF is a more effective therapeutic target remains unknown.

Increasing evidence suggests that small RNA is tumor-specific and cell-specific [16,17] and may be used as a diagnostic marker. The majority of tsRNAs originate from oncogenic stresses including hypoxia, which is consistent with the hypoxic microenvironment of PC [18]. This suggests that suitable tsRNAs production conditions may be inherent in this disease. Lee et al., 2009 discovered that the expression of ‘TRF-1001’ was associated with cell proliferation [19]. Haussecker et al., 2010 revealed that the Ago proteins were related to the tsRNA (named ‘cand45’) [20]. In 2013, the tRNA fragments acting similar to the miRNA in B-cell lymphoma was found by Maute et al. [21]. Balatti V et al., 2016 attested ts-53 and ts-101 reciprocity with Ago and PiwiL2 proteins, and they are down-regulated in lung cancer and chronic lymphocytic leukemia (CLL) [22]. In addition, it has been shown that tRF-03357 significantly promotes the progression of ovarian cancer [23].

Up-frameshift (UPF) proteins are the core of nonsense-mediated mRNA decay (NMD) contained UPF1, UPF2, and UPF3. They were initially identified in genetic screening of *Saccharomyces cerevisiae* and later found to be implicated in NMD of other eukaryotes [24,25]. Among them, UPF1 regulates other pathways including replication-dependent histone mRNA decay (HMD). In the NMD, UPF1 binds to the premature stop codon (PTC) via a translation termination complex bound [26,27]. The NMD can recognize post-transcriptionally aberrant transcripts and mediate their degradation. The UPF1 is the primary regulator of NMD, with intrinsic helicase and ATPase activity [28–30]. Mutations in the UPF1 gene detected in pancreatic adenosquamous carcinoma (ASC) tumors are the first known example of the NMD gene undergoing genetic alteration in human tumors. It is also the first known gene to be selectively mutated in pancreatic ASC tumors. UPF1 mutations promoted the PC progression [31]. Xinke Wang et al. found that LncRNA SNHG6 promotes the development of CRC via targeted modulation of UPF1 [32]. According to Vivek K, UPF1 and SMG7 show an obvious effect on the transcriptome (Raxwal et al. [33]). As identified by Cuicui et al., the UPF1/SNORD52/CDK1 signaling pathway is implicated in the development of hepatocellular carcinoma (HCC) [34].

The gonadotropin-releasing hormone (GnRH) signaling pathway regulates cancer growth and progression [35]. In some human urogenital tract malignancies, including endometrial, ovarian, bladder, and prostate cancers, GnRH and its receptors have been identified as part of the autocrine system that regulates cell proliferation. Besides, GnRH receptor expression has been found in breast and non-reproductive carcinomas, including PC and glioblastoma [36]. This study demonstrates that the target of differentially expressed tRF is primarily implicated in the GnRH signaling pathway; this suggests that tRF may regulate PC progression through this pathway.

We found that tRF-Leu-AAG is differentially expressed in both PC and paracellular tissues; its expression is lofty in PC cells than that in normal PC. *In vitro* experiments revealed that PC cells were promoted by up-regulation of tRF-Leu-AAG on the proliferation, migration, and invasion. Dual-Luciferase Reporter assays indicated that UPF1 is targeted to tRF-Leu-AAG. Additional experiments confirmed that up-regulation of tRF-Leu-AAG could inhibit UPF1 protein levels, demonstrating that UPF1 is negatively regulated by tRF-Leu-AAG. We used functional enrichment analysis to analyze the data to further confirm the functions of target genes, which may be involved in GnRH signaling pathway. Not only further experiments were necessary but we also need to verify their relationship in the organization.

## Conclusion

In conclusion, we report that tRF-Leu-AAG promotes cell proliferation, migration, and invasion through UPF1 down-regulation, thereby promoting PC progression.

## Supplementary Material

Supplemental Material
