# Supplementary material for: The biological behavior of tRNA-derived fragment tRF-Leu-AAG in pancreatic cancer cells
Source: Bioengineered. 2022 Apr 20;13(4):10617–28. doi: 10.1080/21655979.2022.2064206 (PMC9161985; doi:10.1080/21655979.2022.2064206)
Supplement: Supplemental Material [file KBIE_A_2064206_SM2185.zip › supplementary/Table S1.docx]

| **Number** | **Age** | **Sex** | **Tumor Size,cm** | **Tumor Stage** | **Pathological description** |
| --- | --- | --- | --- | --- | --- |
| 1 | 65 | Female | 2.5x2.5x3.0 | T2N1M0 | Middle-differentiated adenocarcinoma invaded nerve, metastasis in 2/7 lymph nodes |
| 2 | 51 | Female | 2.5x2.5x2.5 | T2N2M0 | Middle-differentiated adenocarcinoma invaded nerve and vater, metastasis in 5/11 lymph nodes |
| 3 | 69 | Female | 3.5x3.5x3.0 | T2N0M0 | Middle, low-differentiated adenocarcinoma invaded duodenal wall, no lymph node metastasis |
