# Supplementary figures and images for: The biological behavior of tRNA-derived fragment tRF-Leu-AAG in pancreatic cancer cells
Source: Bioengineered. 2022 Apr 20;13(4):10617–28. doi: 10.1080/21655979.2022.2064206 (PMC9161985; doi:10.1080/21655979.2022.2064206)

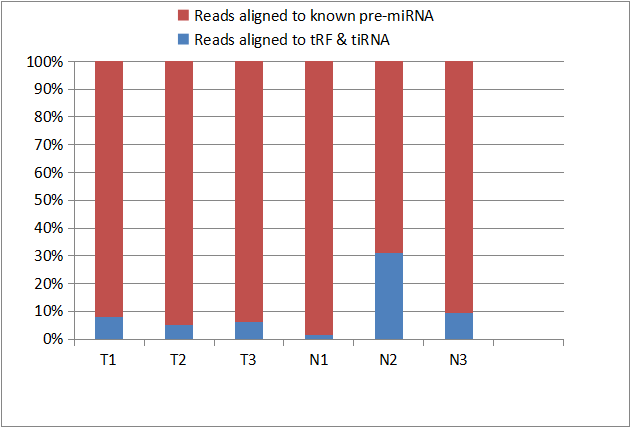

Supplement: Supplemental Material [file KBIE_A_2064206_SM2185.zip › supplementary/Figure S1.tif]
